# Functional expression and properties of the tick α6 homomeric nicotinic acetylcholine receptor in *Xenopus laevis* oocytes

**DOI:** 10.1016/j.crpvbd.2025.100341

**Published:** 2025-12-03

**Authors:** Alison Cartereau, Khalid Boussaine, Emiliane Taillebois, Steeve H. Thany

**Affiliations:** a Université d’Orléans, Laboratoire Physiologie, Ecologie et Environnement (P2E), UR 1207, USC-INRAE 1328, 1 Rue de Chartres, 45067, Orléans, France; b Institut Universitaire de France (IUF), 1 Rue Descartes, 75005, Paris, France

**Keywords:** Tick, *Ixodes ricinus*, ACh, nAChR, Chaperone proteins, Ion channel

## Abstract

Recent studies have suggested that the tick *Ixodes ricinus* expresses nAChR subtypes which are activated by acetylcholine. Here, we investigated the potential of the Iricα6 subunit to form a functional receptor when expressed in *Xenopus laevis* oocytes. Electrophysiological recordings using a two-electrode voltage clamp suggested that the Iricα6 subunit can form a functional homomeric receptor when expressed alone or with chaperone proteins such as RIC-3, UNC-50 and UNC-74. We also found that Iricα6 is a non-selective cation channel. ACh-induced currents were blocked by the nicotinic antagonists methyllicaconitine and dihydro-β-erythroidine. In addition, the nicotinic antagonists α-bungarotoxin and mecamylamine elicited agonist-like responses, with EC_50_ values of 3.48 nM and 12.60 nM, respectively. These data indicated that Iricα6 homomeric receptors could have different pharmacological properties compared to homomeric receptors expressed in other species.

## Highlights

• Iricα6 is orthologous to *Drosophila melanogaster* co-orthologs Dmelα5-6 and 7 subunits.
• Iricα6 subunit was able to form a functional receptor when expressed alone or with chaperone proteins in *Xenopus* oocytes.
• The currents induced by ACh were in accordance with a non-selective cation channel.
• α-bungarotoxin, a selective inhibitor of nicotinic receptors, acted as an activator.

## Graphical abstract

**Figure:**
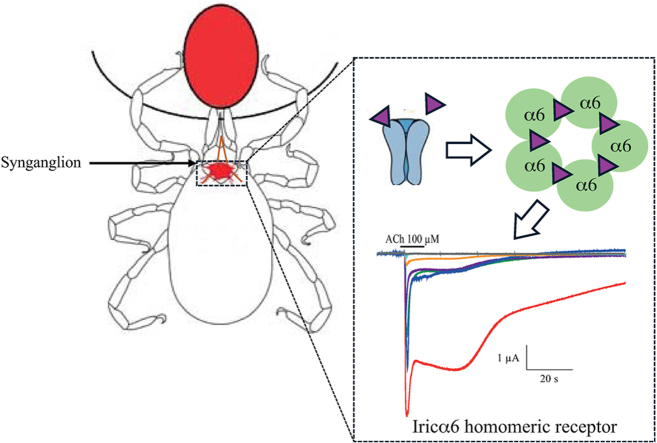

## 1 Introduction

Arthropod nicotinic acetylcholine receptors (nAChRs) are of particular interest. They are involved in rapid neurotransmission and have been identified as contributing to learning and memory function in several insect species, including *Drosophila melanogaster* and the honeybee *Apis mellifera* (Fayyazuddin et al., 2006; Louis et al., 2012). They are also the main target of several compounds used as pesticides, in particular neonicotinoids and recently introduced compounds. Ticks are by far the most economically important ectoparasites of global livestock production. They transmit a variety of pathogens and are hence a major parasite for humans and animals. Despite nAChR subunits having been identified in several tick species, such as the tick *Ixodes scapularis* (Gulia-Nuss et al., 2016) and *Ixodes ricinus* (Rispe et al., 2022), no study has evaluated the functional properties of the tick nAChR subtype. Understanding the functional and pharmacological properties of tick nAChR subtypes is the first step to developing new compounds which can be used for the management strategy against ticks. Indeed, tick control mainly relies on chemical acaricides (George et al., 2004), and resistance has already emerged against every single commercialized acaricide, constituting a global concern for animal health (Abbas et al., 2014). Recently, microtransplantation of tick membranes from the synganglion in *Xenopus laevis* oocytes, and electrophysiological recordings of tick neurons revealed that tick neurons expressed nAChR subtypes which were activated by acetylcholine (ACh) and nicotine (NIC) (Le Mauff et al., 2020; Boussaine et al., 2023). Currents induced by NIC were partially blocked by the nAChR specific antagonist, α-bungarotoxin (α-Bgt), whereas other nAChR antagonists, methyllicaconitine (MLA) and mecamylamine (MECA), did not reduce nicotine-evoked currents (Le Mauff et al., 2020). We hypothesized that tick neurons expressed homomeric nAChR subtypes which can be differently sensitive to α-Bgt and MLA. Indeed, MLA is known as an α7 nAChR antagonist, and it was found that a small proportion of nAChR labelling MLA could be insensitive to α-Bgt (Davies et al., 1999). Transcriptomic analysis performed in the *I. ricinus* synganglion revealed that eight genes coding for nAChR subunits were present in the synganglion, including the Iricα6 subunit (Rispe et al., 2022). Phylogenetic studies have shown that the Iricα6 subunit is the only one orthologous to *Drosophila melanogaster* Dmelα5-6 and 7 subunits (Rispe et al., 2022; Boussaine et al., 2025). We hypothesized that insect α7 orthologs could form homomeric receptors in the same way as mammalian α7 subunits (Thany et al., 2007). Our hypothesis was confirmed by the finding that *D. melanogaster* Dmelα5 and α7 subunits expressed in *X. laevis* oocytes were able to form functional homomeric receptors (Lansdell et al., 2012). In addition, recent studies suggested that insect α6 subunits could form homomeric receptors (Selvam et al., 2015; Hawkins et al., 2022; Lu et al., 2022). Co-expression of *A*. *mellifera* Amelα6 with the chaperone protein NACHO led to a functional homomeric receptor, in which ACh-induced currents were antagonized by α-Bgt (Hawkins et al., 2022). If our hypothesis is true, the Iricα6 subunit, which is in the same cluster as Dmelα6 and mammalian α7 subunits, should also be able to form a homomeric receptor when expressed in a heterologous system such as *X*. *laevis* oocytes.

The first attempt to express a functional tick nAChR subtype in *X. laevis* oocytes was performed for the tick *Rhipicephalus sanguineus* (*sensu lato*) (Lees et al., 2014). The Rsanα1 subunit forms a functional receptor when expressed in *X. laevis* oocytes, but it failed to express homomeric receptors with or without the addition of *Caenorhabditis elegan*s resistance-to-cholinesterase (RIC-3) or *Xenopus laevis* RIC-3 (Lees et al., 2014). Co-expression with the chicken β2 nAChR subunit evoked concentration-dependent inward currents in response to ACh. Interestingly, the hybrid receptor Rsanα1/β2 was insensitive to both the neonicotinoid insecticide imidacloprid (IMI) and the spinosyn, spinosad (Lees et al., 2014). Thus, at present, no studies have been able to demonstrate a functional expression of a tick homomeric nAChR subtype.

In the present study, we evaluated the potential of the tick *I. ricinus* Iricα6 subunit to form a functional receptor when expressed in *X. laevis* oocytes. We found that the Iricα6 subunit was able to form a functional receptor when expressed alone or with chaperone proteins. In addition, under our test conditions, Iricα6 receptors were activated by α-Bgt, a specific inhibitor of homomeric nAChRs.

## 2 Materials and methods

### 2.1 Oocyte sample

Distinct batches of defolliculated *Xenopus laevis* oocytes were purchased from Ecocyte Bioscience (Dortmund, Germany) and Tefor Paris-Saclay (France).

### 2.2 Oocyte injection

Oocytes were injected using Nanoinject II (Drummond Scientific Company, USA) with 10 ng cRNA mix containing Iricα6 cRNA and 5 ng of cRNAs coding for each chaperone protein, according to the previously published protocols (Papke and Porter Papke, 2002; Cartereau et al., 2018; Papke et al., 2018; Cieslikiewicz-Bouet et al., 2020).

### 2.3 Two-electrode voltage clamp

Electrophysiological recordings were made using Roboocyte 2 hardware (Multi Channel Systems MCS GmbH, Germany). Membrane currents were recorded using two microelectrodes filled with 3 M KCl and with a resistance between 0.2 and 5 MΩ (Cartereau et al., 2018). Oocytes were voltage-clamped at −60 mV and perfused with SOS at 3 ml/min (Papke et al., 2018). To inhibit potential muscarinic responses, SOS was supplemented with atropine at 1 μM (Papke and Porter Papke, 2002). Electrophysiological data were acquired using Roboocyte 2 software (Multi Channel Systems MCS GmbH, Germany). The peak current (μA) for each recording was collected at 5 KHz. All recordings were performed at room temperature (21 ± 2 °C).

### 2.4 Reversal potential determination

The reversal potential (E_rev_) of the ACh-induced currents was determined by measuring the peak current at various command potential steps between −100 mV and +60 mV (20 mV step increments). The Na^+^ permeability relative to the K^+^ (P_Na_/P_K_) and the Ca^2+^ permeability relative to Na^+^ (P_Ca_/P_Na_) were calculated using a form of the Goldman-Hodgkin-Katz (GHK) constant field equation as previously used (Barbara et al., 2008).

### 2.5 Compounds

All compounds were purchased from Sigma Aldrich (St Quentin, France), and Fisher Scientific (Illkirch, France). ACh was prepared in water at 1 M and diluted in SOS. 30 nl of 100 mM BAPTA was injected into the oocytes, according to the methods previously used (Haghighi and Cooper, 2000; Bertaud et al., 2024). MECA, α-Bgt, dihydro-β-erythroidine (DHβE), and MLA were prepared in water or DMSO, then diluted in SOS at the tested concentrations. The final DMSO concentration was less than 0.1%. ACh, α-Bgt and MECA were applied for 10 s followed by a 5-min washout period (Papke and Porter Papke, 2002; Cartereau et al., 2018; Gulsevin et al., 2019).

### 2.6 Data analysis

Data were shown as mean ± standard error of the mean (SEM). The EC_50_ values and the statistical analysis of the normalized data were calculated using PRISM 10 (GraphPad Software, La Jolla, CA, USA). Significant differences were calculated using Kruskal-Wallis or Student’s t-test, followed by Bonferroni *post-hoc* test, depending on the size of the group and whether the data were parametric or non-parametric. All data points were included in each assay. The *R*^2^ for each current-voltage curve was determined using the Boltzmann sigmoid equation (PRISM 10, GraphPad Software, La Jolla, CA, USA).

## 3 Results

### 3.1 Expression in *Xenopus laevis* oocytes

**Fig. 1:**
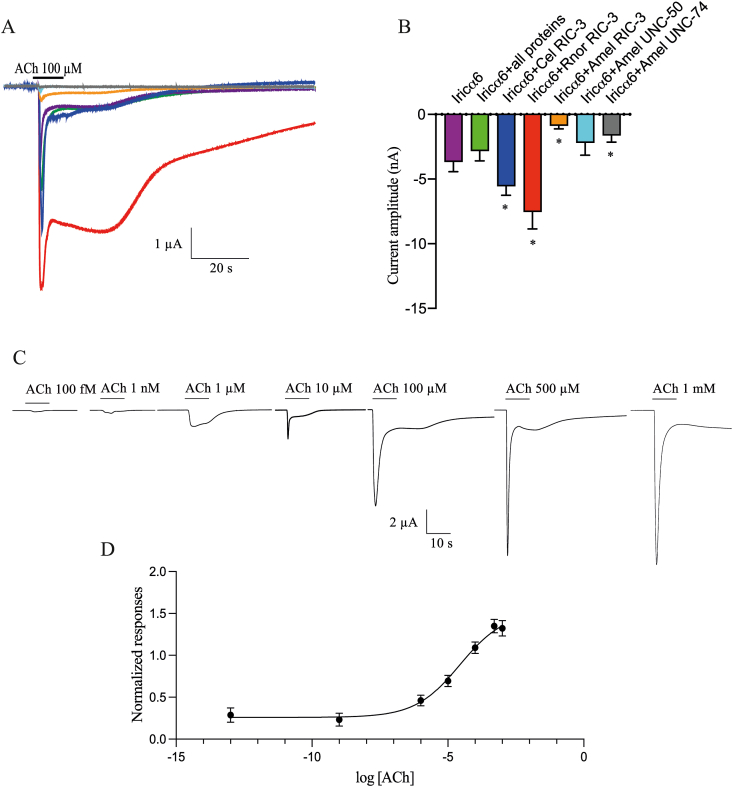
Effect of ACh on the Iricα6 homomeric receptors expressed in *Xenopus laevis* oocytes. **A** Application of 100 μM ACh (10 s application) induced inward currents in which the amplitudes are dependent to the chaperone proteins. **B** Histograms illustrating the mean current amplitudes of 100 μM ACh using each chaperone protein. *Abbreviations*: Cel RIC-3, *C. elegans* RIC-3; Rnor RIC-3, Rattus RIC-3; Amel RIC-3, UNC-50 and UNC-74, *A. mellifera* RIC-3, UNC-50 and UNC-74, respectively. Each histogram represents the mean ± SEM of at least 10 different recordings (*n* = 3–5 different batches of *Xenopus* oocytes. See results part). 100 μM ACh-evoked-current amplitudes were compared using the Kruskal-Wallis test. The asterisk indicates a significant difference with ACh-evoked currents using Iricα6 alone. **C** Typical example of ACh-evoked currents at different concentrations. **D** ACh dose-response curve. Each point represents the mean ± SEM of at least 22 recordings of eight different batches of oocytes.

We first assessed the capacity of Iricα6 cRNA to form functional receptors in *X. laevis* oocytes. As shown in Fig. 1, when Iricα6 cRNA alone was injected into *X. laevis* oocytes, functional receptors were expressed without chaperone proteins. The mean current amplitudes induced by 100 μM ACh were −3.66 ± 0.78 μA (*n* = 22 recordings, using 8 different batches of oocytes, Fig. 1). In previous studies, it was demonstrated that chaperone proteins, such as RIC-3, UNC-50 and UNC-74, increase the functional expression of arthropod nAChRs (Ihara et al., 2020; Brunello et al., 2022). Thus, we assessed the effect of 100 μM ACh with each chaperone protein and compared ACh-evoked current amplitudes. We first found significant differences between current amplitudes. Indeed, our results demonstrated a significant increase in ACh-induced current amplitudes when co-expressed with *Caenorhabditis elegans* RIC-3 (mean current amplitudes of −5.28 ± 0.98 μA, *z* = 3.27, *P* = 0.02, *n* = 10 recordings using 5 different batches), and *Rattus norvegicus* RIC-3 (mean current amplitudes of −7.27 ± 1.58 μA, *z* = 3.32, *P* = 0.02, *n* = 12 recordings using 5 different batches), whereas co-expression with *A*. *mellifera* RIC-3 strongly decreased current amplitudes to −0.86 ± 0.24 μA (*z* = 3.12, *P* = 0.03, *n* = 14 recordings using 4 different batches). Other *A. mellifera* chaperone proteins, such as UNC-50 and UNC-74 decreased ACh-evoked currents to −2.06 ± 0.72 μA (*z* = 2.57, *P* = 0.21, *n* = 10 recordings using 3 different batches) and −1.54 ± 0.41 μA (*P* < 0.05, *n* = 10 recordings using 3 different batches), respectively (Fig. 1). It should be noted that co-injection of Iricα6 with a mix containing all chaperone proteins had no significant difference compared to currents induced by 100 μM ACh, when Iricα6 was injected alone. In the next experiment, for a rapid comparison with the current literature, Iricα6 cRNA was co-expressed with *C. elegans* RIC-3 chaperone proteins. In this context, ACh induced dose-dependent responses with an EC_50_ value of 27.66 ± 2.44 μM.

### 3.2 Iricα6 homomeric receptor and relative ion channel permeabilities

**Fig. 2:**
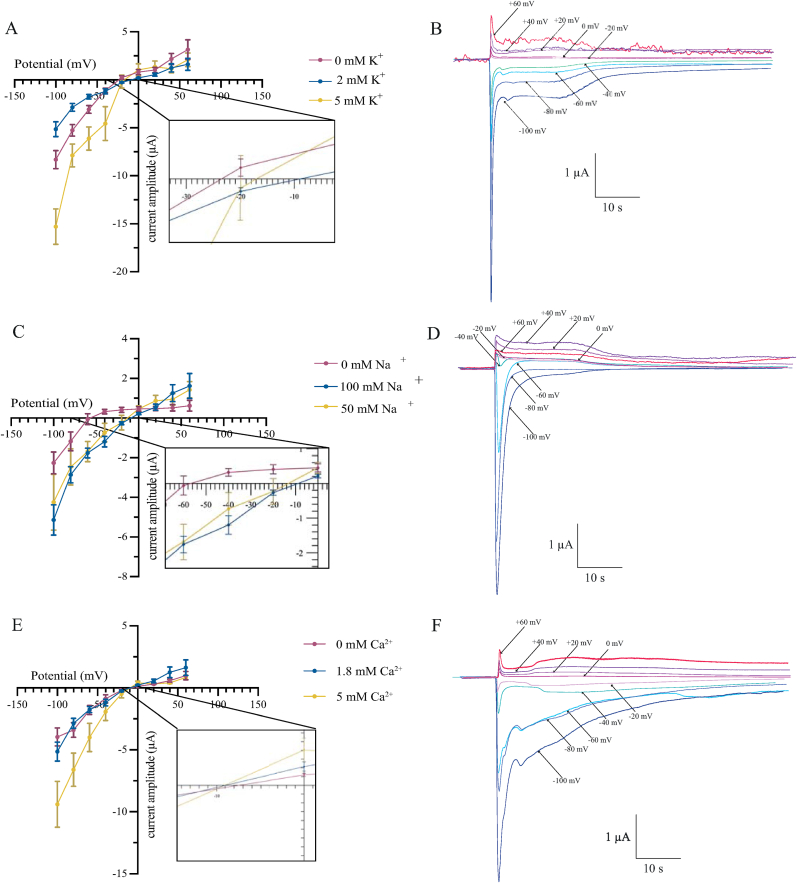
Reversal potential determination of ACh-induced currents in saline solutions containing different K^+^, Na^+^ and Ca^2+^ ion concentrations. In the left the current-voltage curves, and in the right, examples of the ACh-induced currents at membrane potential from −100 mV to 60 mV by increments of 20 mV. Insets show the E_rev_ for each ion. **A**, **B** The ACh-elicited currents reversed at −23.5 ± 1.7 mV and −17.0 ± 0.9 mV, at 0 mM and 5 mM, respectively. **C**, **D** ACh-induced currents reversed at −56.7 mV and −13.4 mV, for 0 mM and 50 mM Na^+^, respectively. **E**, **F** For Ca^2+^, ACh-induced currents reversed at −9.0 ± 3.6 mV and −7.5 ± 1.8 mV at 0 mM and 5 mM, respectively. For all experimental conditions, each point represents at least *n* = 12 recordings, from 4 different batches of oocytes. In accordance with the Boltzmann sigmoid equation, *R*^2^_K_^+^ = 0.87, *R*^2^_Na_^+^ = 0.76, and *R*^2^_Ca_^2+^ = 0.92.

**Fig. 3:**
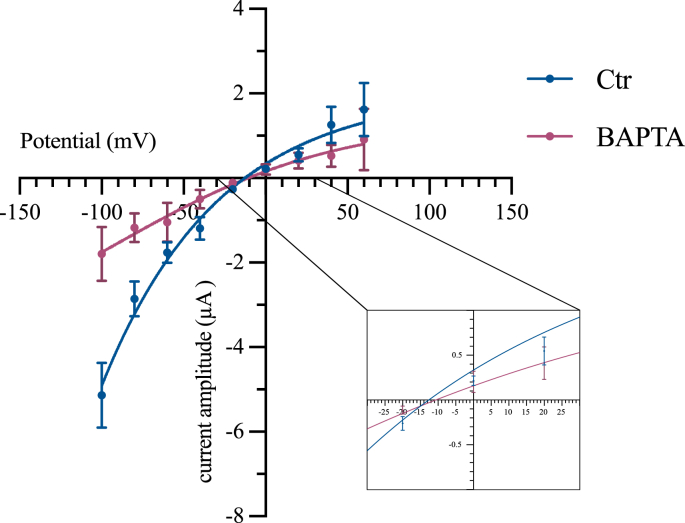
Current-voltage curve under intracellular application of BAPTA. Determination of E_rev_ (−13 ± 2.6 mV), using intracellular application of 100 mM BAPTA into the oocytes. Mean values ± SEM are plotted. Each point represents *n* = 10 recordings from at least 3 different batches of oocytes. *Abbreviation*: Ctl, standard saline (see material and methods). In accordance with the Boltzmann sigmoid equation, *R*^2^ = 0.88.

To characterize potential modification of Iricα6 channel selectivity, we performed a series of measurements of E_rev_ in the presence of various Na^+^, K^+^ and Ca^2+^ concentrations. It is important to note that we do not use single-channel recordings; therefore, the E_rev_ values should be considered in the context of overall currents. We first found that the current-voltage relationship was linear from −100 mV to +60 mV. The currents induced by ACh had E_rev_ at −12.05 mV (Fig. 2), in accordance with a possible non-selective cation channel. To validate this assumption, we estimated the contribution of the Na^+^, K^+^ and Ca^2+^ influx to the total ACh-induced currents. As shown in Fig. 2, increasing the K^+^ concentration of the external saline (2 mM K^+^ in standard saline) to 5 mM or decreasing to 0 mM, shifted the E_rev_ to −23.5 ± 1.7 mV and −17.0 ± 0.9 mV, and increased the ACh-evoked current amplitudes between −100 mV and −20 mV (*P* < 0.05, *n* = 12 oocytes, Fig. 2A and B). Similarly, decreasing the Na^+^ concentration from 100 mM (in standard saline) to 50 mM had no effect on the E_rev_ or current amplitudes. Removing Na^+^ from the external saline reduced the peak current amplitudes by 50% and shifted the E_rev_ to −56.7 ± 2.3 mV (*n* = 12 oocytes, Fig. 2C and D), suggesting that Na^+^ was involved in the ACh-evoked currents. An estimate of the Na^+^ to K^+^ permeability ratio (P_Na_/P_K_) under these ionic conditions was found using the GHK constant field equation. The best agreement with experimental data was obtained when P_Na_/P_K_ = 1.23. To determine whether Iricα6 homomeric receptors are permeable to Ca^2+^, we increased external Ca^2+^ concentration from 1.8 mM (standard saline) to 5 mM. The E_rev_ shifted to −7.5 ± 1.8 mV (*n* = 12 oocytes, Fig. 2E and F). Decreasing Ca^2+^ concentration to 0 mM shifted the E_rev_ to −9.0 ± 3.6 mV. The P_Ca_/P_Na_ ratio was estimated as 0.95. Intracellular Ca^2+^ concentration was suppressed by injecting 30 nl of the Ca^2+^ chelator BAPTA (100 mM) into the oocytes (Galzi et al., 1992; Cens et al., 1997). As shown in Fig. 3, E_rev_ was at −13 mV when using 100 mM BAPTA (Haghighi and Cooper, 2000; Bertaud et al., 2024), but the ACh-evoked currents significantly decreased at hyperpolarizing potentials (*n* = 10 oocytes, *P* < 0.05).

### 3.3 α-bungarotoxin and mecamylamine responses at the tick a6 nicotinic acetylcholine receptors

**Fig. 4:**
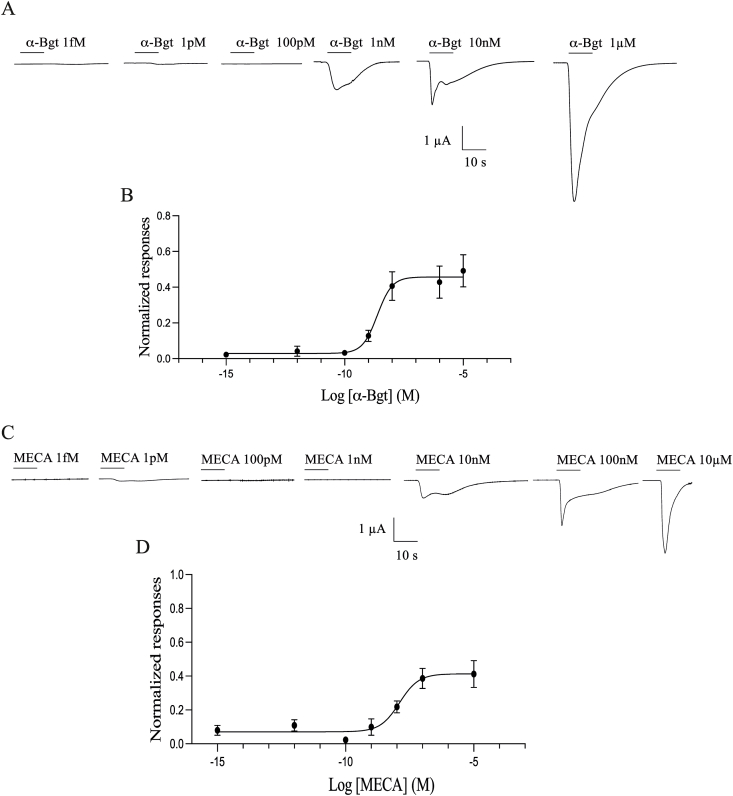
Agonist effect of α-Bgt and mecamylamine on Iricα6 homomeric receptors. **A** Currents induced by α-Bgt which did not induce any current at a concentration lower than 100 pM. **B** Dose-response curve. **C** MECA-evoked currents on Iricα6 homomeric receptors expressed in the oocytes. MECA at a concentration of 1 nM did not induce any current. **D** Dose-response curve. Data for each point were normalized to the peak amplitude of the response at 100 μM ACh. Mean values ± SEM were plotted. In all experimental conditions, each point represents *n* = 14 oocytes from 4 different batches.

**Fig. 5:**
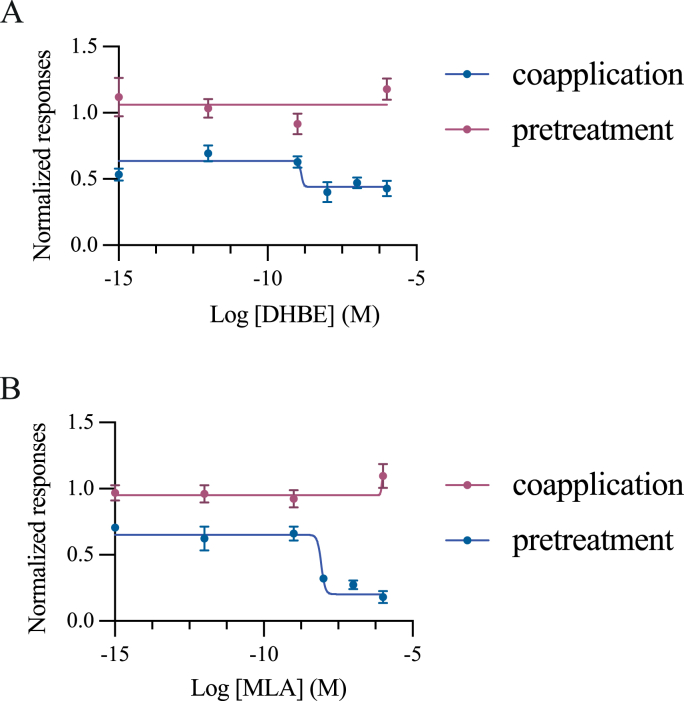
Inhibitory effects of dihydro-β-erythroidine and methillycaconitine on ACh-evoked currents. Inhibition curves of ACh-induced currents with the nicotinic antagonists dihydro-β-erythroidine (DHβE) (**A**) and methyllicaconitine (MLA) (**B**). As found, MLA did not induce a complete inhibition of ACh-evoked current amplitudes. Peak current amplitudes induced by 27.66 μM ACh (EC_50_-value) during the antagonist application were measured and normalized to the current elicited before the antagonist application. Each point represents the mean ± SEM of *n* = 14 oocytes from 4 different batches.

The pharmacological properties of Iricα6 homomeric receptors were investigated using several nAChR antagonists superfused as pre-application and coapplication with 100 μM ACh as indicated in Section 2. Fig. 4 shows that α-Bgt acted as an activator of Iricα6 receptors. Indeed, it induced a dose-response curve with an EC_50_ value of 3.48 nM (*n* = 14 different recordings, using 4 different batches of oocytes, Fig. 4A). Similarly, the nicotinic antagonist, MECA, induced a dose-response curve with an EC_50_ value of 12.60 nM (*n* = 14 different recordings, using 4 different batches of oocytes, Fig. 4B). Given these responses, we wanted to evaluate two other antagonists, MLA and DβHE. MLA is a specific inhibitor of homomeric nAChR, and DβHE is an inhibitor of heteromeric nAChR subtypes. However, 100 nM MLA inhibited ACh-evoked currents, as found with 100 nM DβHE, when coapplied with 100 μM ACh (*n* = 14 different recordings, using 4 different batches of oocytes, Fig. 5A and B). The IC_50_ values were 8.56 nM and 1.35 nM (*n* = 12 different recordings, using 4 different batches of oocytes in each experimental condition), respectively. We also noted that MLA and DβHE did not elicit agonist-like responses, as found with α-Bgt and MECA (data not shown).

## 4 Discussion

The functional expression of arthropod neuronal nAChRs has been somewhat a challenge, and had only been resolved for some subunits, particularly in *D*. *melanogaster* (Lansdell and Millar, 2004; Lansdell et al., 2012), and more recently, the honeybee *A. mellifera* (Ihara et al., 2020; Brunello et al., 2022). In the present study, we demonstrated a functional expression of the Iricα6 receptor in *X. laevis* oocytes, suggesting that this subunit can form an ion channel. It was demonstrated that α6 subunits, which form a cluster with the human α7 subunit, form functional homomeric receptors when expressed in *X. laevis* oocytes. Unfortunately, no previous studies have evaluated the electrophysiological properties of arthropod α6 homomeric receptors. Using voltage steps, the E_rev_ of ACh-evoked currents indicated that the Iricα6 is a non-selective cationic channel. Indeed, the relative Na^+^, K^+^ and Ca^2+^ permeabilities of the Iricα6 receptor suggested that the shifts were not sufficient to consider a selective permeability for one of the ions. Our results were close to those found using honeybee neurons, demonstrating that P_Ca_/P_Na_ and P_Ca_/P_K_ were around 0.54 and 0.87, respectively (Barbara et al., 2008). Moreover, mammalian α7 homomeric receptors, which are highly permeable to Ca^2+^, have a P_Ca_/P_Na_ ratio of approximately 20 in *X. laevis* oocytes, which is greater than any of the other nAChRs (Seguela et al., 1993; Wallace and Porter, 2011). However, caution must be taken in our study because of the possible activation of Ca^2+^-activated chloride (Cl^−^) channels. Additional experiments will be done to separate the activation of the Iricα6 channel from the possible subsequent activation of the Ca^2+^-dependent Cl^−^ channels. Our data also demonstrated that inhibiting intracellular Ca^2+^ concentrations with BAPTA reduced ACh-evoked current amplitudes, consistent with the possibility that Ca^2+^ could regulate the Iricα6 homomeric receptors. This finding is in accordance with our previous study, which demonstrated that tick neurons from the synganglion display an increase in intracellular calcium levels after pulse application of ACh (Boussaine et al., 2023). Similar observations were found in mammalian nAChRs. Calcium can regulate the α7 homomeric nAChRs *via* Ca^2+^-binding sites (Galzi et al., 1996).

In all cases, our results suggested that the Iricα6 subunit can form robust functional receptors when expressed alone, suggesting that chaperone proteins are not a necessary or sufficient condition to express a functional Iricα6 homomeric receptor in *X. laevis* oocytes. Indeed, it was suggested that co-factors, such as RIC-3 and NACHO, are necessary to enable robust expression of insect nAChRs in *X. laevis* oocytes (Lansdell et al., 2012; Ihara et al., 2020). RIC-3 chaperone proteins act to pull individual pre-folded α7 nAChR subunits through the dimerization of its two coiled-coiled domains or assist in the folding of subunits prior to assembly. NACHO requires the α7 ectodomain to promote receptor assembly and surface trafficking (Wang et al., 2009; Kweon et al., 2020). However, it seems that NACHO-mediated assembly is independent and separate from that of RIC-3 (Kweon et al., 2020). We found that the RIC-3 chaperone proteins from *C. elegans* and *R. norvegicus* increased ACh-evoked current amplitudes, whereas chaperone proteins from the honeybee *A*. *mellifera* strongly decreased current amplitudes. The way in which these RIC-3 proteins act to assist the folding and assembly of Iricα6 receptors was not evaluated in the present study. However, intrinsically, RIC-3 presents little sequence homology across species. The most plausible hypothesis in our study is that some chaperone proteins could lead to a lack of functional expression or a reduction in current amplitudes when co-expressed with Iricα6 subunit. Thus, because Iricα6 subunit is expressed in mammalian heterologous system, RIC-3 from *C. elegans* and *R. norvegicus* could enhance the functional expression of Iricα6 homomeric nAChR.

Another important point which seemed to differentiate the Iricα6 from mammalian α7 homomeric receptors was that both α-Bgt and MECA acted as activators of Iricα6 receptors. MLA, however, was an inhibitor. α-Bgt is a specific inhibitor of mammalian homomeric receptors (daCosta et al., 2015), and it was demonstrated that α7 receptors expressed in the oocytes were highly sensitive and inhibited by nanomolar concentrations of α-Bgt (Couturier et al., 1990; Cuevas and Berg, 1998). The binding properties of α-Bgt and MECA to the Iricα6 homomeric receptors are unclear at this stage. We cannot exclude the possibility that they can act as partial agonists, or that in our experimental conditions, the results are due to a desensitisation of the Iricα6. However, previous studies suggested that α-Bgt acts as an agonist in hybrid nAChRs containing *Drosophila* α subunits (Schulz et al., 2000), and *Nilaparvata lugens* Nlα2/β2 and Nlα1/Nlα2/β2 receptors (Liu et al., 2009). Overall, while our findings suggest that Iricα6 homomeric receptors could have specific pharmacological properties against currently used nAChR antagonists, additional studies are needed to confirm their pharmacology.

## 5 Conclusions

In the present article, we focus mainly on the molecular expression of the Iricα6 subunit in *X. laevis* oocytes and its activation by ACh. Further concerted efforts are necessary to effectively exploit Iricα6 as a drug target for tick management. The first step will be to continue studying the pharmacological properties of Iricα6. The functional expression of Iricα6 without chaperone proteins is intriguing and could suggest that tick nAChRs present specific folding and assembly properties in *X. laevis* oocytes. It will be interesting to see whether other tick nAChR subtypes have the same functional expression. The second step will be to study its binding affinities to currently used acaricides, insecticides and repellents targeting arthropod nAChRs. Indeed, several compounds targeting insect nAChRs were used as insecticides and repellents. Nevertheless, they are poorly developed against tick species because of (i) resistance mechanisms and (ii) insufficient studies focusing on understanding the functional and pharmacological properties of tick nAChR subtypes. The cloning, functional and pharmacological properties of Iricα6 receptors will enable these compounds to be tested and will potentially open the way to understanding the mechanisms of acaricide resistance. Indeed, studies have demonstrated the importance of the arthropod α6 subunits in the toxic effects of pesticides such as spinosyn (Urena et al., 2019; Zeng et al., 2024; Mocchetti et al., 2025). Thus, it will be interesting to compare the binding properties of spinosyn with other pesticides targeting arthropod nAChRs, such as neonicotinoids, sulfoximines and butenolides.

## Ethical approval

Not applicable.

## CRediT authorship contribution statement

Alison Cartereau: Formal analysis, Investigation, Data curation, Writing - original draft, Writing – review & editing. Khalid Boussaine: Formal analysis, Investigation. Emiliane Taillebois: Formal analysis, Data curation, Writing - original draft. Steeve H. Thany: Conceptualization, Methodology, Validation, Writing - original draft, Writing – review & editing, Supervision, Funding acquisition.

## Funding

This study was supported by funding from the Region Centre-Val de Loire, under Electrocell funding. K. Boussaine received a PhD grant from the ANR Axotick.

## Declaration of competing interests

The authors declare that they have no competing financial interests or personal relationships that could have appeared to influence the work reported in this paper.

## Data Availability

The data that support the findings of this study are available from the corresponding author upon request.
